# Research‐ and health‐related youth advisory groups in Canada: An environmental scan with stakeholder interviews

**DOI:** 10.1111/hex.13316

**Published:** 2021-07-19

**Authors:** Michelle Chan, Shannon D. Scott, Alyson Campbell, Sarah A. Elliott, Hannah Brooks, Lisa Hartling

**Affiliations:** ^1^ Faculty of Nursing University of Alberta Edmonton Alberta Canada; ^2^ Department of Pediatrics, Faculty of Medicine and Dentistry, Alberta Research Centre for Health Evidence University of Alberta Edmonton Alberta Canada; ^3^ Faculty of Nursing, ECHO Research University of Alberta Edmonton Alberta Canada

**Keywords:** advisory group, engagement, environmental scan, health, qualitative interviews, research, youth

## Abstract

**Background:**

Engaging youth throughout the research process improves research quality and outcomes. Youth advisory groups provide one way for youth to express their opinions on relevant issues.

**Objective:**

This study aimed to identify research‐ and health‐related youth advisory groups (‘groups’) in Canada and understand the best practices of these groups.

**Methods:**

Google searches and supplementary methods were used to identify relevant groups in Canada. Group information was extracted from websites or through interviews with key informants.

**Results:**

We identified 40 groups. Groups were commonly part of a hospital/healthcare facility, nonprofit/health organization or research group. The majority focused on a specific content area, most commonly, mental health. Over half the groups advised on health services. Members' ages ranged from 9 to 35 years. The number of members ranged from 5 to 130. Interviews (*n* = 12) identified seven categories relating to group practices: (a) group purpose/objectives, (b) group development, (c) group operations, (d) group structure, (e) adult involvement, (f) membership and recruitment and (g) group access. Challenges and facilitators to the success of groups were described within the following themes: (a) retaining engagement, (b) creating a safe environment and (c) putting youth in positions of influence. Advice and recommendations were provided regarding the development of a new group.

**Conclusion:**

This study provides a comprehensive overview of research‐ and health‐related youth advisory groups in Canada. This information can be used to identify groups that stakeholders could access as well as inform the development of a new group.

**Patient or Public Contribution:**

Youth advisory group representatives were interviewed as part of the study.

## 1 BACKGROUND

Over the last decade, there has been a growing reconceptualization of young people in health and research contexts. Young people are now more than ever actively participating in setting research agendas, codesigning research studies, informing knowledge translation activities^1^ and acting in an advisory capacity to researchers and policy makers.^2^, ^3^, ^4^, ^5^ Empowering young people to share their unique and valuable experiences can improve research quality and relevance by ensuring that research projects and outputs (e.g., knowledge translation tools) align with the needs and perspectives of youth.^6^, ^7^, ^8^

Youth engagement has been defined as ‘the meaningful and sustained involvement of a young person in an activity focused outside the self’.^9^ One approach to engaging young people is through youth advisory groups. Such advisory groups (either general or population/patient‐specific) allow youth to be included as partners offering advice and feedback on issues that affect them, rather than simply acting as participants in the process.^2^, ^10^ In Canada, there is emerging literature on the development of youth advisory groups in research and health.^2^, ^11^, ^12^, ^13^, ^14^ For example, Ramey et al.^14^ describe a youth advisory group focused on youth health issues. The group is involved in decision‐making, advocacy and coordination of their own projects. Evaluation of the group identified positive relationships between youth and adult allies, opportunities for new perspectives on youth priorities and opportunities for skill development. Various models have been proposed to conceptualize youth engagement in research.^2^, ^13^, ^15^ One that has been successfully applied in a Canadian context is the McCain Model of Youth Engagement.^13^ The model outlines the varying levels at which youth can be engaged in research projects based on their interests, skills and availability.

While the value of incorporating the patient/consumer voice in research is well established, there is little practical guidance for researchers looking to work with youth in an advisory capacity. Our research programme is focused on improving health outcomes for children through knowledge translation. As such, we are interested in understanding how best to work with an existing youth advisory group (if available) to provide input on our research activities and/or develop a new group to support our work.

To facilitate this, a comprehensive search to identify and provide information on research‐ and health‐related youth advisory groups is needed. Environmental scans (ESs) are a method of gathering information about current and emerging issues through a systematic search of websites and other sources.^16^, ^17^ Additionally, Internet searches are increasingly being used in health research to collect and organize information.^18^, ^19^, ^20^ The objectives of our study were, therefore, to conduct an ES to (1) identify research‐ and health‐related youth advisory groups (referred to herein as groups) in Canada and (2) better understand the structure and functioning of these groups, including best practices (i.e., recommended approaches for successful youth engagement).

## 2 METHODS

This study involved two phases: First, the Internet and supplementary search methods were used to identify relevant groups; second, interviews were conducted with key informants to obtain more information about the identified groups.

### 2.1 Procedures

#### 2.1.1 Phase 1: Internet and supplementary search

Two independent searches were conducted using Google's Advanced Search function between January and February 2020 by A. C. The first search aimed to find groups using the following search terms: *(child OR youth) (health OR research) (advisory) (club OR group OR council OR network OR committee)*. After finding a small number of results that were specifically research or health related, we expanded our second search by removing *(health OR research)*. The first 100 website URLs from each search string were reviewed.^21^ The search was limited by region to ‘Canada’ only. The search terms were developed through examination of peer‐reviewed literature and consultation with the research team.

Screening and full review of each website were conducted concurrently. The following inclusion criteria were applied: (a) members include children/youth (<35 years); (b) the group is research or health related; (c) the group is based in Canada; and (d) the group has an advisory role (defined as a group of individuals that provides advice and feedback on important issues for an organization or research team). We limited our search to Canada as one of our objectives was to identify groups that our research programme could access to support its activities in a Canadian context. To supplement the internet search, we hand‐searched reference lists of relevant articles, contacted relevant organizations to ask if they were affiliated with a group and to identify others and consulted key informants from relevant groups (Phase 2).

#### 2.1.2 Phase 2: Key informant interviews

The Internet search was complemented by interviewing key informants at relevant organizations identified from the search. Attempts were made to contact informants from 15 organizations whose mandate and activities most aligned with the work of our research programme (Table 1). Key informants were identified as those who currently co‐ordinate the group and/or work at an organization overseeing the group. Informants were contacted about the study via email or telephone, with a follow‐up within 1–2 weeks if there was no response. Upon agreement to participate, a phone or online interview was scheduled at a convenient time for the informant. Participants provided written, informed consent before the interview. Ethics approval was granted by the University of Alberta Health Research Ethics Board (Pro00098715).

**Table 1:** Youth advisory groups contacted for key informant interviews

Alberta Children's Hospital Child and Youth Advisory Council
BC Children's Hospital Youth Advisory Group
CHEO Youth Forum
City of Lethbridge Youth Advisory Council
ErinoakKids Youth Advisory Committee
Holland Bloorview Youth Advisory Council
Human Environments Analysis Laboratory Youth Advisory Council
London Health Sciences Centre Children's Hospital Child and Youth Advisory Council
Métis Nation BC Métis Youth Mental Health and Wellness Initiative (formerly Métis Mental Health Youth Advisory Committee)
MICYRN KidsCan Young Persons' Advisory Group
SickKids Children's Council
Stollery Youth Advisory Council

### 2.2 Data collection

#### 2.2.1 Phase 1: Internet and supplementary search

Data were extracted by one author (A. C.) into Excel spreadsheets. Predetermined group attributes were collected (i.e., group name, location, number of members, etc). A second author (M. C.) verified the extracted data. Attempts were made by M. C. to contact groups with no website (identified from supplementary search) or little information on their website for further information.

Each group was assigned a level of engagement based on their group activity information extracted from websites and/or through key informant interviews. Levels of engagement were based on the International Association for Public Participation (IAP2) spectrum. The IAP2 spectrum differentiates between five levels of engagement in decision‐making processes: Inform, Consult, Involve, Collaborate and Empower.^22^ The spectrum has been used in various countries including Canada;^23^ therefore, we adapted it to describe the different ways in which youth can be involved in decision‐making (Table 2 provides the definitions).

**Table 2:** Level of youth engagement^a^

Level	Definition
Inform	The group receives information from the organization, government or research team to assist in understanding the problem
Consult	The group provides feedback on analysis, options and/or decisions
Involve	The group works directly with the organization, government or research team throughout the process (i.e., research process, service design process, policy development process, etc.) rather than only at a particular stage
Collaborate	The group works in partnership with the organization, government or research team in each aspect of the decision
Empower	The group makes final decisions for the organization, government or research team

*Note*: Adapted from the International Association for Public Participation spectrum of public participation. https://www.iap2canada.ca/Resources/Documents/0702-Foundations-Spectrum-MW-rev2%20(1).pdf.

^a^ Engagement in this context is defined as the process of involving youth in problem‐solving or decision‐making and using youth input to make better decisions.

#### 2.2.2 Phase 2: Key informant interviews

Qualitative interviews were conducted following an interview guide (Supporting Information Appendix A). Interview questions focused on information that could not be obtained by reviewing the website (e.g., how the group operates, how the group was developed). Members of the research team trained in qualitative data collection (A. C., M. C.) conducted the interviews individually. Interviews were audio‐recorded and transcribed verbatim by a professional transcriptionist.

### 2.3 Data analysis

#### 2.3.1 Phase 1: Internet and supplementary search

Data describing each group were presented in summary tables. Where appropriate, descriptive statistics were used.

#### 2.3.2 Phase 2: Key informant interviews

Interview data were uploaded to NVivo 12 qualitative data management software and analysed using content and thematic analyses by M. C. Transcripts were read in detail several times. Phrases in the text were identified and codes were developed to represent key concepts. The codes were then grouped into categories and themes.^24^ A second coder (A. C.) verified the coding, categories and themes in a random subset of interview data (25%),^25^ with no disagreements. Rigour was ensured by using strategies such as an audit trail, reflexivity and immersion in the data, as suggested by Lincoln and Guba,^26^ to achieve credibility, dependability, confirmability and transferability.

## 3 RESULTS

### 3.1 Phase 1: Internet and supplementary search

**Figure 1:**
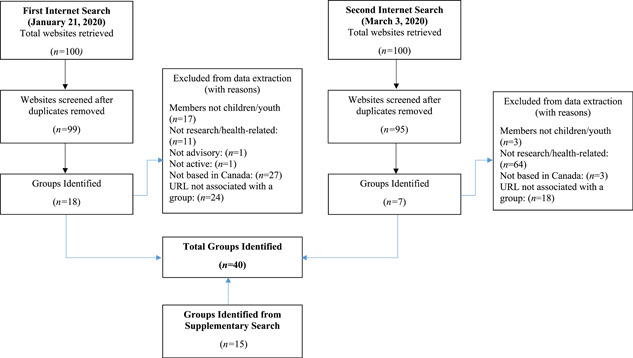
Flow diagram of the search strategy

One hundred websites were identified from each Internet search (200 websites in total). After removing duplicates, 194 websites remained and were screened. Twenty‐five groups fulfilled the inclusion criteria (Figure 1). An additional 15 groups were identified from the supplementary search method, for a total of 40 groups included. The characteristics of the included groups are shown in Table 3.

**Table 3:** Characteristics of research‐ and health‐related youth advisory groups (*n* = 40; listed in alphabetical order by group name)^a^

Group name	Central location (level of operation)	Year established	Number of members	Age of members	Membership criteria	Group interest^b^	Type of advisory work^c^	Level of youth engagement^d^	Group meetings (format)
ACCESS Open Minds National Youth Council^e^	Montreal, Quebec (national)	2014	NK	16–30	Lived experiences with mental health challenges and services	B (mental health)	1, 2	Collaborate	Bimonthly
Alberta Children's Hospital Child and Youth Advisory Council	Calgary, Alberta (regional)	2003	25–30	12–21	Current or former patient or sibling/family member of a patient at Alberta Children's Hospital, the Child and Adolescent Addiction and Mental Health Programme or other healthcare facilities; live with a disability or chronic health condition; lived experience with adversity	A	2, 3	Consult	Six times/year, whole day, Saturday (in‐person, online^b^)
BC Children's Hospital Youth Advisory Group	Vancouver, British Columbia (local)	2010	12–20	12–23	Current or former patient or sibling of a patient at BC Children's Hospital	A	2, 3	Consult	Monthly, 2 h, evening (in‐person, online^b^)
Canadian Mental Health Association National Youth Advisory Council	Toronto, Ontario (national)	2019	10–15	15–30	General youth in Canada	B (mental health)	1, 2, 3, 4	Involve	Teleconference
Centre for Addiction and Mental Health McCain Centre Youth Advisory Group	Toronto, Ontario (local)	2016/2017	20	16–29	Lived experience with mental health and/or substance use challenges	B (mental health and substance use)	1, 2	Collaborate	Monthly (in‐person, online^b^)
Centre for Addiction and Mental Health National Youth Action Council	Toronto, Ontario (national)	2014	130	16–25	General youth in Canada	B (mental health and substance use)	1, 2, 3, 4	Collaborate	NK
CHEO Youth Forum	Ottawa, Ontario (local)	2000	19	12–18	Current or former patient or sibling of a patient at CHEO	A	2, 3	Consult	Monthly, 2 h, evening (in‐person, online^b^)
CHEO YouthNet/RéseauAdo Youth Advisory Committee	Ottawa, Ontario (local)	NK	NK	13–20	General youth from the community	B (mental health)	2, 4	Involve	Weekly, 2 h, evening (in‐person, online^b^)
CHILD‐BRIGHT National Youth Advisory Panel	Quebec (national)	2018	6	NK	Lived experience with a brain‐based developmental disability	B (brain‐based disabilities)	1	Consult	NK
CIHR IHDCYH Youth Advisory Council	Halifax, Nova Scotia (national)	2020	12	12–25	General youth in Canada, passionate about health and/or health research	A	1, 3	Involve	Quarterly (online, at least one in‐person meeting/year)
City of Edmonton Youth Council Health and Wellness Committee	Edmonton, Alberta (local)	NK	21	13–23	General youth in Edmonton	A	1, 3, 4	Consult	NK
City of Lethbridge Youth Advisory Council	Lethbridge, Alberta (local)	2017	11	14–25	High school student in Lethbridge, postsecondary student in Lethbridge, community youth representative	A	1, 4	Consult	Monthly, 2h, evening (in‐person, online^b^)
ErinoakKids Youth Advisory Committee	Mississauga, Brampton, Orangeville, and Halton, Ontario (regional)	1989	5–8	14–19	Current or former patient at ErinoakKids	B (disabilities)	2	Consult	Monthly, 1–1.5 h (in‐person, online^b^)
Food Allergy Canada Youth Advisory Panel	Toronto, Ontario (national)	NK	60+	13–25	Lived experience with a food allergy	B (food allergies)	2, 4	Consult	NK
Foundry's Provincial Youth Advisory Committee^e^	British Columbia (provincial)	NK	NK	NK	NK	B (mental health)	2	NK	NK
Frayme Advisory on Youth Matters	Ottawa, Ontario (national)	NK	NK	18–29	Lived experience with mental health and/or substance use challenges	B (mental health)	2	Consult	NK
Health Canada Youth Leadership Team (formerly Youth Action Committee)	Ottawa, Ontario (national)	1999	NK	16–22	General youth in Canada	B (tobacco use)	2	Consult	NK
Holland Bloorview Youth Advisory Council	Toronto, Ontario (local)	1996	24	14–29	Current or former patient at Holland Bloorview; independently mobile	B (disabilities)	2	Consult	Monthly, 2 h, Saturday (in‐person, online^b^)
Hôtel‐Dieu Grace Healthcare Youth Advisory Council	Windsor, Ontario (local)	2017	14	15–19	General youth from the community	A	2	Consult	Bimonthly (online, one in‐person meeting/year)
Human Environments Analysis Laboratory (HEAL) Youth Advisory Council	London, Ontario (local)	2018	16	13–19	High school student in London	A	1	Collaborate	Biweekly, 2 h, evening (in‐person, online^b^)
IWK Youth Advisory Council	Halifax, Nova Scotia (Maritimes)	2006	NK	13–18	Current or former patient or sibling of a patient at IWK	A	2, 3	Consult	Monthly, 2 h, evening
Kids Help Phone National Youth Council	Toronto, Ontario (national)	NK	15	14–24	General youth in Canada, passionate about mental health	B (mental health)	4	Involve	Monthly, 2 h (online)
KidsAbility Youth Advisory Council	Waterloo Region and Guelph‐Wellington, Ontario (regional)	2017	NK	14–24	General youth in Waterloo Region or Guelph‐Wellington	B (disabilities)	2	Consult	Monthly, 1.5 h (in‐person and online)
London Health Sciences Centre Children's Hospital Child and Youth Advisory Council	London, Ontario (local)	2010	14	12–20	Current or former patient or sibling of a patient at London Children's Hospital	A	2	Consult	Monthly, 1.5 h, evening (in‐person, online^b^)
McCreary Youth Advisory & Action Council	Vancouver, British Columbia (provincial)	1995	15–20	15–24	General youth in British Columbia	A	1, 4	Involve	NK
Mental Health Commission of Canada Youth Council	Ottawa, Ontario (national)	2008	8	18–30	Lived experience with mental health challenges, personally or through a friend/family	B (mental health)	1, 2, 3, 4	Involve	NK (Teleconference, at least one in‐person meeting/year)
Métis Nation BC's Métis Youth Mental Health and Wellness Initiative (formerly Métis Mental Health Youth Advisory Committee)	British Columbia (provincial)	2019	15	14–35	Metis youth in British Columbia	B (mental health; Metis community)	1, 4	Consult	Monthly (online, one in‐person meeting/year)
MICYRN KidsCan Young Persons' Advisory Group	Vancouver, British Columbia (national)	2012 (local), 2017 (national)	15	13–19	Current or former patient treated at a paediatric healthcare facility in Canada	A	1	Consult	Monthly, 1–2 h, Saturday (teleconference/online)
Mood Disorders Society of Canada National Youth Advisory Council	Belleville, Ontario (national)	2019	14	18–25	Lived experience with mental health challenges, personally or through a friend/family	B (mental health)	2, 3, 4	Consult	Quarterly (online)
The New Mentality and Children's Mental Health Ontario's Youth Action Committee	Toronto, Ontario (provincial)	2012	9	16–25	NK	B (mental health)	2, 3	Collaborate	Monthly (conference calls, 3–4 in‐person meetings/year)
NorthBEAT Youth Advisory Group	Northwestern Ontario (regional)	2018	40+	12–25	Lived experience with mental health services, personally or through a friend/family	B (mental health)	2	Consult	Monthly or bimonthly, 1 h
NorWest Co‐op Community Health Youth Advisory Committee	Winnipeg, Manitoba (provincial)	NK	NK	14–25	General youth in Manitoba	B (mental health)	2	Consult	Monthly, 1 h (in‐person, online^b^)
Ontario Centre of Excellence for Child & Youth Mental Health Youth Advisory Council	Ottawa, Ontario (provincial)	2019	12	NK	General youth in Ontario	B (mental health)	1, 2, 3	Involve	NK
Pathstone Mental Health Youth Advisory Committee	Niagara Region, Ontario (regional)	NK	NK	14–25	General youth in the Niagara Region	B (mental health)	2, 4	Consult	NK
The Sandbox Project's Young Canadians Roundtable on Health	Toronto, Ontario (national)	2013	32	15–30	General youth in Canada	B (injury prevention, mental health, growing healthy bodies, environment)	1, 2, 3, 4	Empower	NK
SickKids Children's Council	Toronto, Ontario (local)	2000	15	9–18	Current or former patient or sibling of a patient at SickKids	A	2, 3	Consult	Monthly, 2 h, evening (in‐person, online^b^)
Stollery Youth Advisory Council	Edmonton, Alberta (national)	2016 (local), national NK	28	12–25	Current or former patient or sibling of a patient at the Stollery Children's Hospital	A	2, 3	Consult	Six times/year, whole day, Saturday (in‐person and online, online only^b^)
Youth Mental Health Canada Youth Advisory Group	Hamilton, Ontario (NK)	2018	NK	NK	NK	B (mental health)	4	NK	Monthly or bimonthly (in‐person)
Youth Wellness Hubs Ontario's Provincial Youth Advisory Council^e^	Ontario (provincial)	2019	20	NK	Lived experience with mental health and/or substance use services	B (mental health)	2	Collaborate	NK
YouthCan IMPACT Youth Advisory Group	Toronto, Ontario (local)	2016	NK	14–24	Lived experience with mental health and addiction challenges/services	B (mental health and addiction)	1, 2	Involve	Bimonthly

Abbreviation: NK, not known.

^a^ See Supporting Information Appendix B for the groups' website URLs.

^b^ Group interest A: General, B: Specific (focus).

^c^ Type of advisory work: (1) research, (2) health programmes/services, (3) health‐related policies; and (4) health promotion.

^d^ See Table 3 for definitions.

^e^ The organization has additional local youth advisory groups at individual centres/sites.

#### 3.1.1 Group association

Affiliations of the 40 groups included hospital or healthcare facility (*n* = 14, 35%); research or knowledge mobilization centre, group or network (*n* = 8, 20%); nonprofit or health organization (*n* = 10, 25%); integrated youth services initiative (*n* = 4, 10%; integrated youth services bring together service providers to create accessible, youth‐friendly, integrated hubs for mental health, substance use and related issues); city government (*n* = 2, 5%); collaborative initiative of researchers and service providers (*n* = 1, 3%); and federal government (*n* = 1, 3%).

#### 3.1.2 Group location and level of operation

The level of operation for one group was not known. Of the other 39 groups, most operated nationally (*n* = 14, 36%) or locally (*n* = 12, 31%). Seven groups operated provincially (18%), five regionally (13%) and one interprovincially (3%).

#### 3.1.3 Year established

Amongst the groups with known year of establishment (*n* = 32), the majority were established between 2010 and 2020 (*n* = 24, 75%). In comparison, only seven groups were established earlier, between 1989 and 2009. The median year of establishment was 2015.

#### 3.1.4 Group interest

Over half of the groups (*n* = 26, 65%) focused on a specific content area, with mental health being the most common (*n* = 19, 48%). Other areas were disabilities (*n* = 3), brain‐based disabilities (*n* = 1), tobacco use (*n* = 1) and food allergy (*n* = 1). One group focused on issues of injury prevention, mental health, growing healthy bodies and the environment (*n* = 1).

#### 3.1.5 Type of advisory work

All groups provided advice and feedback on initiatives related to at least one of the following categories: research, health programmes/services, health policies and health promotion. Over half of the groups (*n* = 24, 60%) conducted work in at least two categories. Advising on health programmes/services and health policies was the most common combination (*n* = 7, 18%). The majority of groups (*n* = 30, 73%) provided feedback on health programmes/services for children/youth within their organization or at a system level. A total of 16 groups (40%) advised on research‐related activities, 15 (38%) on health policies and 14 (35%) on health promotion activities. Four groups (10%) conducted work in all four categories.

#### 3.1.6 Group members

For 11 groups, the number of members was unclear. Among the other 29 groups, the number of members ranged from 5 to 130, with the majority having between 10 and 20 members (*n* = 17, 61%). Groups with more than 30 members were national or regional (*n* = 4, 14%). The age of members ranged from 9 to 35 years. For five groups, members' ages were not reported.

Of the 37 groups with known membership criteria, 10 (27%) required members to be a patient (or sibling of a patient) treated at a hospital or healthcare facility. Another common criterion for membership was specific lived experience (e.g., mental health challenges, disabilities; *n* = 10, 27%). Other requirements for membership included being a high school student (*n* = 1), a high school student/postsecondary student/community youth representative (*n* = 1) and Metis youth (*n* = 1). For 14 groups (38%), members were made up of youth in general (with or without lived experiences) and/or youth who were passionate about the group's area of focus.

#### 3.1.7 Group meetings

Of the 26 groups with known information on *frequency of meetings*, the majority met monthly (*n* = 15, 58%). Other meeting frequencies were bimonthly (*n* = 3), six times/year (*n* = 2), four times/year (*n* = 2), weekly (*n* = 1) and biweekly (*n* = 1). Two groups met monthly or bimonthly.

Of the 13 groups with available information on *meeting times*, the majority met on weekday evenings (*n* = 9, 69%) and 4 met during the day on Saturdays (31%). Of the 17 groups with available information on *meeting duration*, 10 (59%) had meetings that were 2 h long and 5 (29%) had meetings that were 1–1.5 h long. Two groups (13%) had meetings for a whole day on Saturdays.

Of the 24 groups with known information on *meeting format*, over half (*n* = 13, 57%) typically met in‐person, nine (38%) typically met online via video/teleconference and two (9%) met in‐person with video/teleconferencing options. Due to the COVID‐19 pandemic, 12 groups (50%) were known to shift from meeting in‐person to online.

#### 3.1.8 Level of youth engagement

The level of youth engagement was unclear for two groups. Of the remaining 38 groups, 23 (61%) were engaged at the level of Consult, 8 (21%) at Involve, 6 (16%) at Collaborate and 1 (3%) at Empower.

### 3.2 Phase 2: Key informant interviews

A total of 12 interviews were completed with 13 group representatives and two youth members. Seven categories relating to group practices were identified: (1) group purpose/objectives, (2) group development, (3) group operations, (4) group structure, (5) adult involvement, (6) membership and recruitment and (7) access to the group. Themes around challenges and facilitators to the success of groups were as follows: (1) retaining youth engagement, (2) creating a safe environment and (3) putting youth in positions of influence. Key informants also put forward advice and recommendations regarding the development of a new group. See Table 4 for data that highlight and bring life to the qualitative analysis.

**Table 4:** Quotes from key informants illustrating the themes and categories

Themes and categories	Example quotes
Group practices	
1. Group purpose/objectives	“…the purpose of the council is to provide a youth perspective on decisions made in the hospital that will be affecting youth and children patients…” (Y‐003)
	“…to integrate youth perspectives into youth health research.” (Y‐012)
2. Group development	“There was a lot of shared literature and shared practices primarily based out of the UK and Europe that was very helpful in helping us start and form the foundations of the national Canadian group.” (Y‐001)
	“The first thing we did was we did a literature review of other [youth advisory] councils…. and then after that … we started interviewing other [youth advisory] councils and youth experts in [the city].” (Y‐012)
3. Group operations	“People that run a program or service, looking to change a policy or a procedure or anything to that effect, would generally come to our group and consult with us asking, ‘What do
	you think? What would you like to see changed? Do you think this is a good program? Would there be uptake?'” (Y‐010)
	“…[a] researcher contacted us and wanted [the youth advisory group] to go over a survey tool that they were developing for use with children and youth…” (Y‐001)
	“Primarily our meetings are consultations…. we have a lot of researchers that come in contact with the group for advice or…consultation” (YAG‐001)
	“…[at meetings] we discuss topics that are brought to us from directors of departments and leaders and … give feedback on [projective/initiatives] that they bring to us.” (YAG‐003)
4. Group structure	“We thought that the more [staff] roles you put in, the more [the youth] feel like they're not contributing to the discussion as much.” (Y‐012)
5. Adult involvement	“…it ends up being a little more intimidating and the dynamic of the group does shift a little bit once there's a really large number of adults in the room.” (YAG‐010)
6. Membership and recruitment	“Members are recruited through … [the research group's] Instagram page and Twitter.” (YAG‐012)
	“We have posters up in the units … with information in a QR code… Also, a lot of the physicians are aware of the work of the [youth] council and if they have a patient who they think would be a good fit, then they will bring
	it up with the patient and suggest that they look into it.” (YAG‐003)
	“…we try to reflect the diversity of youth in the community. We try and have people from different demographic backgrounds… [and with] different experiences.” (YAG‐009)
7. Access to the group	“I meet with the people coming and bringing consults to [the youth advisory group]. I meet with them one to one [to] find out what it is they're looking for.” (YAG‐002)
	“We are always looking to collaborate, especially in research, and I know the youth love having a voice in the bigger picture of things.” (YAG‐003)
Challenges and facilitators to the success of youth advisory groups	
1. Retaining youth engagement	“I find sometimes it's a little bit challenging to take research work and make it engaging…” (Y‐002)
	“Reviewing policies and procedures … can be pretty boring. If I don't manage to find engaging ways of doing [these activities], some [youth] leave and that's what had happened in the first two years.” (YAG‐010)
2. Creating a safe environment	“The youth know each other so there's a real comfort level and a feeling of safety in expressing their opinions and their thoughts.” (Y‐002)
	“The honesty and the openness within the group really lends well to [its success] and the fact that they feel that this is a non‐judgemental place.” (Y‐010)
3. Putting youth in positions of influence	“For every meeting, two members would sign up for that meeting to be co‐Chairs. They would co‐Chair the meeting together and lead the whole meeting…” (Y‐010)
	“I'm there to help support [the youth advisory group], but as much as possible, we want them [the youth] to be running and making the decisions for council.” (Y‐015)
Advice/recommendations for developing a new group	“…one huge takeaway is youth are youth. They have their own lives, they have their own interests, and they are not the same as adults. There are a number of considerations that have to be kept in mind when … [engaging] with youth.” (YAG‐008)
	“…make sure there is informed preparation for each of your consultations…. A lot of the times what happens is [the] youth are not adequately prepared before they come to the consultation table and so they don't participate as fully themselves…” (YAG‐001)

### 3.3 Categories related to group practices

#### 3.3.1 Group purpose/objectives

The purpose of many groups was similar and typically revolved around providing a *youth perspective* on research‐related activities or aspects of healthcare that have an impact on children and youth. Youth were often involved in providing advice and feedback for stakeholders, rather than making final decisions for them. While the primary purpose of most groups was to serve in an advisory capacity to their organization or research, they participated in other activities as well, such as leading their own projects and events.

#### 3.3.2 Group development

The development process of groups varied. Two groups examined the models of other youth groups in hospitals and other organizations. One group was modelled after an existing youth advisory group in the same province. One group conducted an informal literature review on youth participation and other youth advisory groups and consulted with local youth experts and stakeholders with experience in youth advisory groups. The foundations of one group were informed by practices based out of youth advisory groups in Europe. One group was informed by the literature on grassroots community development and youth participation. Three key informants stated that their group was developed in partnership with youth. Due to their recent involvement with their group, four key informants could not accurately speak to their group's development process.

#### 3.3.3 Group operations

Almost all groups operated by providing advice and feedback on research‐related activities or aspects of health service planning/policy development/health promotion through a consultation process with stakeholders. Consult requests typically came from researchers, hospital or healthcare facility leadership, healthcare providers and/or other staff.

All groups had regular meetings. Meetings were held at convenient times (evenings or Saturdays) and followed the academic year (September–June). Parking and public transit expenses were generally reimbursed for youth attending meetings in‐person. Groups operating locally or regionally typically had their meetings in‐person. Provincial and national groups typically met online, with at least one in‐person meeting a year. Zoom was the most common platform for online meetings.

Meetings were often Chaired or co‐Chaired by youth members who self‐selected to be in the positions. Chairs typically served for one year. Chairs' responsibilities were to lead and facilitate meetings and assist with creating the agenda (with staff). For three groups, the Chair positions were rotated on a monthly basis.

While the specifics of the meeting structure differed for each group, the pattern was typically consistent. Groups often had a meeting agenda that was sent out ahead of time. Meetings started with introductions and/or icebreakers, followed by updates within the organization or research group. During consultation meetings, members heard from a guest speaker seeking youth input on a project/initiative. Members then shared their thoughts and opinions through roundtable discussions or informal focus groups. During general (nonconsultation) meetings, members discussed projects/events that they were involved in.

Many key informants described their meetings to be informal, open and honest. In some groups, members shared ideas and opinions using sticky notes on the wall, drawings and/or flip charts to make meetings more engaging. All groups had food available. Many incorporated socializing times at their meetings for team bonding. Most groups expected members to attend a minimum number of meetings each year, ranging from 50% to 100% of meetings. For two groups, flexibility was key and there was never any pressure for members to attend meetings.

Email was the most common method of communication between staff and youth members outside of the scheduled meetings. Four groups used other platforms to communicate with members including group texting (*n* = 1), Facebook Messenger (*n* = 1), Instagram (*n* = 1) and Basecamp (*n* = 1). Aside from Basecamp, key informants said that these platforms were what youth were already accessing and were comfortable with. Communications outside of meetings were on an as‐needed basis, ranging from no communication for 1–2 weeks to several in a day.

#### 3.3.4 Group structure

Almost all groups had a small organizational structure with one or two part‐time staff coordinators/facilitators. Staff were typically adults associated with the organization. For two groups, staff were youth themselves. Staff provided support in terms of meeting coordination, logistics and liaising with stakeholders. One group had a larger and more hierarchical organizational structure with a patient engagement co‐ordinator (responsible for connecting with stakeholders and bringing consult requests to the group), three staff facilitators and a leadership team (made up of five youth members who represented the group and worked alongside the staff facilitators).

#### 3.3.5 Adult involvement

Almost all groups had minimal adult involvement. Aside from guest speakers, parents of the youth and other adults were not involved in any advisory capacity and did not attend meetings. Some key informants said that keeping the adult presence/voice out was important to ensure that members felt comfortable and free to express their thoughts.

#### 3.3.6 Membership and recruitment

All key informants said members in their groups were volunteers and received no monetary compensation for their time; however, key informants acknowledged that other groups may provide compensation. All groups offered the opportunity to receive references and volunteer hours. Members generally served a 1‐year term, with the opportunity for an additional year(s) for those who wanted to remain in the group.

Many groups aimed to recruit a diverse group of youth varying in age, gender, culture, ethnicity, geographic location, socioeconomic status and lived experience. Key informants acknowledged the importance of diversity among youth and ensuring that a broad range of perspectives and experiences are brought to the table. Groups often recruited new members over the spring and/or summer. The most common methods of recruitment were via online advertising (social media, websites), posters on bulletin boards in hospitals/clinics and promotion through hospital staff and physicians. For one group, recruitment was entirely through nomination by hospital staff rather than through public advertising.

Half of the groups did not have a formal application and/or interview process for interested members. Six groups required youth to fill out an application if they were interested in joining the group. Of these, five conducted an interview with their applicants.

Most groups did not offer formal training/orientation for new members. However, groups that focused on research were more likely to provide training/orientation on topics such as research methods, ethics and the basics of patient‐oriented research.

#### 3.3.7 Access to the group

Almost all groups accepted requests from external researchers or organizations to access their group; however, internal requests were typically prioritized. To access most groups, stakeholders were to contact the staff co‐ordinator and they would bring the project/initiative to leadership for approval and/or schedule a meeting to discuss further. One group required stakeholders to complete a formal council engagement request form. Requests were often screened for appropriateness and fit and scheduled by the staff co‐ordinator. For two groups, external requests were accepted or declined by youth members through general consensus.

### 3.4 Themes related to facilitators and challenges around engaging youth

#### 3.4.1 Creating a safe environment

Creating a safe environment for youth was the most common facilitator to the success of groups. Key informants commented on the value of having a safe space where youth feel respected, included, supported and comfortable enough to engage.

#### 3.4.2 Putting youth in positions of influence

Many key informants noted the success of their group resulted from giving members leadership opportunities. For many groups, acting in an advisory capacity was only part of the group's activities. Members also led their own projects and events. These activities were all youth‐driven. Staff supported the youth, but allowed them to lead the process. Key informants said that it was important to allow members to come up with ideas and make decisions.

#### 3.4.3 Retaining youth engagement

Many key informants highlighted that retaining youth engagement and interest was a challenge. Four noted that some members in their group did not attend meetings regularly, which reduced member attendance and made meetings less engaging. One suggested that the lack of compensation may be a disincentive for members to participate. Another key informant spoke of the difficulties with engaging members across different geographic areas. Some key informants spoke of the challenges around ensuring that projects/initiatives are relevant and appealing to members.

### 3.5 Advice/recommendations for developing a new group

Several key informants said that it was important to have a clear idea of what the group's purpose and goals are from the outset. Key considerations for effective youth participation included ensuring that the meeting time is accessible for youth, having food available at meetings, ensuring that youth have a safe space to speak freely and ensuring that projects/initiatives that members are advising on are of interest and engaging to them. Some key informants said that it was important to consider the number and type of adults and/or staff involved; minimal adult/staff involvement was recommended. Other advice included ensuring that youth are adequately prepared before each consultation meeting, ensuring diversity of members by reaching specific communities or groups of people, developing relationships with similar groups, ensuring that members have the appropriate training in research (if advising on research) and receiving feedback from members early on in the process as opposed to later.

## 4 DISCUSSION

This study aimed to identify and provide a comprehensive overview of research‐ and health‐related youth advisory groups in Canada. The purpose of many groups was to provide a youth perspective on research‐ and/or health‐related activities that have an impact on children and youth. The literature shows that hearing from youth on issues that affect them is the best way to identify priorities and ensure that processes and outcomes reflect the needs and views of youth.^6^, ^7^

From the ES, we identified 40 groups across Canada that engage youth in research‐related activities or various aspects of health service planning/policy development/health promotion across a spectrum of health topics. Many of the groups were recently established (between 2010 and 2020), reflecting an increase in the awareness of youth engagement as an important part of research‐ and health‐related improvement processes^3^, ^27^ This aligns with the growing body of literature in the last decade that has noted a shift in youth engagement in research, planning and policy development.^4^, ^5^, ^27^, ^28^

Mental health was the most common area of focus, with almost half of the groups in our scan advising on mental health‐related research projects and programmes/services within their organization and/or at a system level. This is not surprising as the literature in the last decade has called for an increase in youth engagement in mental health research^29^ and service planning.^27^, ^30^ People with lived experience of mental health issues are crucial to shaping mental health research and services because they have ‘been there’ and can offer insights and suggestions that may not otherwise come to light.^29^, ^31^, ^32^ Unsurprisingly, many of the mental health groups included in this ES recruited youth with lived experience.

### 4.1 Age and group dynamics

Interestingly, the age of group members varied widely, ranging from 9 to 35 years. Currently, there is no universal definition of youth. For instance, ‘youth’ is defined as ages 15–24 by the United Nations,^33^ 15–29 by Statistics Canada^34^ and 16–40 by the National Youth Policy of Nepal.^35^ The literature suggests that age differences can influence group dynamics and collaboration processes as ‘younger youth’ may think and engage differently than ‘older youth’ due to differences in age, maturity, cognitive capacity and experiences.^36^, ^37^ Within an advisory group, wide age disparities could cause divergence in priorities and input, as well as distort power relations within the group (i.e., the 15‐year‐old may view the 35‐year‐old as an adult rather than a peer).

It is also interesting to consider whether age should matter at all in youth engagement. In Western societies, youth is often defined by age; however, in some countries, youth and adulthood are defined by ‘a distinct social status with specific roles, rituals, and relationships’,^38^ such as getting married and having children. Thus, the sociocultural environment, which varies among cultures, can define when one is a youth. This may contribute to exclusion when recruiting youth from different cultures or societies where individuals aged 30 or older may be seen as too old to participate in youth groups; however, they may consider themselves young because they have not met the cultural markers of adulthood. Another important consideration of including older youth is that they can provide input of lived experience during their younger years.

### 4.2 Level of youth engagement

The majority of groups were engaged at the Consult level (61%) on the IAP2 spectrum, followed by Involve (21%). At both levels, the goal is to listen to members in the group and acknowledge their feedback. The next two levels, Collaborate and Empower, exemplify more intensive levels of engagement. While powerful, they can be more challenging to implement. Only six groups (16%) were placed at the level of Collaborate, where the goal is to work in partnership with the group and share the decision‐making power. Developing a collaborative group requires more time, resources and commitment from members, which may explain why fewer groups are engaged at this level.^39^ Only one group (3%) was considered to be at the Empower level (Young Canadians Roundtable on Health), where the group makes final decisions for the organization. This level of influence is rarely seen as most organizations do not have the authority to delegate decision‐making to external stakeholders.^39^ A publication on the YCHR describes a unique model where the group is largely independent of the organization and is given authority to make decisions without having to obtain approval by the organization.^14^ Our findings are similar to Crockett et al's.^23^ study on health researchers' experiences in engaging patients and the public in health research. Among 53 Manitoban health researchers in Crockett et al's^23^ study, most reported engaging with patients and the public at the levels of Inform (81.3%), Consult (64.6%) or Involve (54.2%), while fewer engaged at the levels of Collaborate (37.5%) or Empower (12.5%). We note that our findings only reflect youth engagement in an advisory group capacity and that, outside of advisory groups, youth may be engaged in more extensive manners by the organization (e.g., as staff, research partners).

### 4.3 Considerations for developing a youth advisory group

#### 4.3.1 Involve youth from the beginning

Our findings show that it is important to involve youth in the development of the group. Engaging youth in the development process ensures that the group is relevant to their unique needs and provides opportunities for youth leadership. This was highlighted by the Human Environments Analysis Laboratory Youth Advisory Council, who involved youth in every step of the development process^2^ and reported high levels of engagement and enthusiasm from participating youth.

#### 4.3.2 Ensure that youth are engaged and empowered

To foster engagement, many groups kept the group size manageable (10–20 members, with one or two staff coordinators) and addressed the power imbalance between youth and adults by limiting the number of adults involved. This aligns with the literature suggesting that it is best practice to engage youth and adults separately, such as having distinct youth and family advisory groups, to ensure that the youth voice is not overpowered by adults.^1^

Many groups adopted a youth‐friendly approach to meetings by setting a casual tone and encouraging youthful styles of working that were social and interactive (e.g., writing down ideas on sticky notes and placing them on a wall, incorporating socializing time at meetings).^40^ Much of the literature on youth engagement in research, health settings and the community provides recommendations for the creation of youth‐friendly spaces that are safe and welcoming for youth.^1^, ^41^, ^42^

Youth members often chaired and facilitated meetings, as well as led their own projects and events. Findings show that when youth are given more autonomy and responsibility, they become more engaged and motivated in what they do.^40^ The literature also speaks to the multiple benefits associated with providing youth with leadership opportunities.^8^ In research, when youth see other youth in leadership positions (e.g., facilitating discussions), it may build their confidence to share ideas and increase participation.^1^

Creamer et al.^43^ found similar challenges with respect to maintaining members' attendance and engagement in a mental health promotion group for young people. Hawke et al.^1^ recommend providing some kind of formal recognition to young people so that they know that their time and contributions are being valued; this could be in the form of wages, an honorarium, references, letters of support and/or certificates. van Schelven et al.^28^ also suggested reimbursing young people involved in research to increase commitment to projects.

#### 4.3.3 Training and information needs

A gap was revealed in terms of training/orientation for new members. A common barrier cited by group leaders was a lack of access to educational training materials. Tsang et al.^3^ found that the amount of training received by youth advisory groups focused on research varied. The authors suggest a need for standardized protocols for the training of youth on research processes. On the other hand, some literature cautions against overtraining members to the point where they ‘lose their grassroots experiential perspectives’^44^ and no longer represent the public.^4^

### 4.4 Accessing youth advisory groups

Almost all groups were willing to provide access to their group by external researchers or organizations, which questions the need to form a new youth group. The process of developing a new group, from the background research to launch, takes time and resources in terms of staff, finances and processes. Thus, tapping into an existing group may be more efficient and cost‐effective. Another benefit is that it may offer researchers and organizations more flexibility, as they can access different groups according to specific needs. However, the disadvantage to accessing an existing group is the potential for delays in waiting for access. Many groups work on a project‐by‐project basis and leadership and/or staff must review and approve the project before it can be put forward to the group. In most cases, internal projects/initiatives are prioritized over external ones.

### 4.5 Limitations

While this ES used a comprehensive and systematic process, there are limitations due to the lack of methodological guidance for this study method. It is possible that relevant groups were missed as information on the Internet may not be accurate and/or up to date. While we interviewed 15 key informants from 12 organizations, they may not be representative of all groups included in the scan. It is noteworthy that we only reached out to groups that were most relevant to helping our research programme understand how youth engage in research/health‐related processes. Further, the groups identified in this scan are based in Canada, excluding some groups that may be relevant, but located in other countries. Finally, we did not include youth in the design or conduct of this study; this would have added an additional perspective on this topic and should be a consideration for future work in this area.

## 5 CONCLUSIONS

As evidenced by the current number of groups, there is a growing recognition that youth are important stakeholders in research and healthcare. This study provides a comprehensive overview of research‐ and health‐related youth advisory groups in Canada. Our findings provide insight into what groups currently exist, including their purpose, structure, operations and best practices. Considerations, as well as facilitators and challenges, for how best to engage youth were also presented. This information could be used to identify groups that researchers and other relevant stakeholders could access, as well as inform others interested in developing a research‐ and/or health‐related youth advisory group.

## CONFLICT OF INTERESTS

The authors declare that there are no conflicts of interest.

## AUTHOR CONTRIBUTIONS

All authors contributed to the conception and design of the study. Michelle Chan and Alyson Campbell conducted the internet and supplementary searches, extracted the data and analysed the data. Michelle Chan and Alyson Campbell conducted the interviews. Michelle Chan analysed the interview data. Michelle Chan developed the initial draft of the manuscript. All authors reviewed, read and approved the final manuscript.

## Supporting information

Supporting information.

Supporting information.

## Data Availability

The data that support the findings of this study are available on request from the corresponding author. The data are not publicly available due to privacy or ethical restrictions.
