# Supplementary material for: Research‐ and health‐related youth advisory groups in Canada: An environmental scan with stakeholder interviews
Source: Health Expect. 2021 Jul 19;24(5):1763–79. doi: 10.1111/hex.13316 (PMC8483214; doi:10.1111/hex.13316)
Supplement: Supplementary file 1 — Supporting information. [file HEX-24-1763-s002.docx]

**Appendix A.** Key informant interview guide

1. What is your role with the youth advisory group?
2. Can you provide some background information about the youth advisory group?

- When did the group start? Why was the group started? Does the group operate locally, provincially or nationally, and has this changed over time?

1. What is the purpose of the group?
2. Does the group have Terms of Reference? (ask if it can be shared/emailed to you)
3. What kind of activities does the group do? Can you provide an example of what the group has done over time?
4. What informed the structure and activities of the group? Was the group modelled after another group? Was any literature used to inform the group?
5. Who leads or organizes the group?
6. Who decides on the activities of the group and how are these decisions made?
7. What resources are required to maintain the group? For example, staff, space, etc.

- Are additional staff involved? How many? Who are they?

1. How many members are currently in the group?
2. What is the age range of members?
3. What are the membership criteria?
4. How long do members serve? How many terms can members serve?
5. What is the expected time commitment from members?
6. How are members recruited? How often are new members recruited? Is there a screening process (e.g., interview) for recruitment?
7. Are members offered any orientation or training prior to joining? If so, what does this consist of?
8. Are members provided any honorarium/incentives for participating? If so, what does this consist of?
9. How often and how long are meetings?
10. What format is used for meetings (e.g., in-person, video conference, teleconference)?

- If in-person, probe where meetings are held.
- If video/teleconference, probe what kind of technology is used.

1. Is there a Chair? If so, what is their role? How are they selected? How long do they serve?
2. How are meetings run? Who coordinates them? What does a typically meeting look like?
3. Are there communications outside of the structured meetings? If so, how are these communications handled and who does them?

- How often is information communicated to members? What platforms are used for communications?

1. What is the level of youth involvement? For example, do members provide advice only or are they also involved in decision-making?

- If members are involved in decision-making, how are decisions made?

1. Are parents of the youth (or other adults) involved in any way? If so, how are they involved?
2. Has any of the work done by the group been published?
3. What is working well with the group?
4. What are some barriers and challenges with the group?
5. What were some unexpected consequences/experiences that came out of the group?
6. Based on your experiences, what advice would you give to someone starting up a research/health-related youth advisory group?
7. If ECHO/ARCHE wanted to access the youth advisory group to support its research program and activities, would that be possible and what would be the process?
8. Do you know of any other research/health-related youth advisory groups in Canada or relevant contacts you could share?
