# Supplementary material for: Research‐ and health‐related youth advisory groups in Canada: An environmental scan with stakeholder interviews
Source: Health Expect. 2021 Jul 19;24(5):1763–79. doi: 10.1111/hex.13316 (PMC8483214; doi:10.1111/hex.13316)
Supplement: Supplementary file 2 — Supporting information. [file HEX-24-1763-s001.docx]

**Appendix B.** Internet URLs of research and health-related youth advisory groups

| **Group Name** | **URL** |
| --- | --- |
| ACCESS Open Minds National Youth Council | https://accessopenminds.ca/national-youth-council |
| Alberta Children’s Hospital Child and Youth Advisory Council | http://fcrc.albertahealthservices.ca/youth/cayac |
| BC Children's Hospital Youth Advisory Group | http://www.bcchildrens.ca/our-services/clinics/youth-health-clinic |
| Canadian Mental Health Association National Youth Advisory Council | https://cmha.ca/wp-content/uploads/2019/08/National-youth-advisory-council-Background-info.pdf |
| Centre for Addiction and Mental Health McCain Centre Youth Advisory Group | No website |
| Centre for Addiction and Mental Health National Youth Action Council | https://www.camh.ca/en/science-and-research/institutes-and-centres/the-margaret-and-wallace-mccain-centre-for-child-youth-and-family-mental-health/youth-engagement-initiative |
| CHEO Youth Forum | https://www.cheo.on.ca/en/get-involved/youth-forum.aspx |
| CHEO YouthNet/RéseauAdo Youth Advisory Committee | http://ynra.ca/youth-engagement |
| CHILD-BRIGHT National Youth Advisory Panel | https://www.child-bright.ca/youth-mandate |
| CIHR IHDCYH Youth Advisory Council | https://cihr-irsc.gc.ca/e/51876.html |
| City of Edmonton Youth Council Health and Wellness Committee | https://www.ceyc.ca/healthandwellness |
| City of Lethbridge Youth Advisory Council | https://www.lethbridge.ca/City-Government/Boards-Commissions-Committees/Pages/Youth-Advisory-Council.aspx |
| ErinoakKids Youth Advisory Committee | https://www.erinoakkids.ca/About-Us/Accountability/Committees/Youth-Advisory-Committee.aspx |
| Food Allergy Canada Youth Advisory Panel | https://foodallergycanada.ca/our-impact-advocacy-and-services/programs-and-services/youth-advisory-panel-yap |
| Foundry’s Provincial Youth Advisory Committee | No website |
| Frayme Advisory on Youth Matters | https://frayme.ca/news/frayme-advisory-youth-matters-aym-%e2%80%93-targeted-call |
| Health Canada's Youth Leadership Team (formerly Youth Action Committee) | https://www.canada.ca/en/health-canada/services/health-concerns/tobacco/youth-zone/youth-engagement.html / http://nbatc.ca/2019/10/02/health-canada-recruiting-for-youth-leadership-team |
| Holland Bloorview Youth Advisory Council | https://www.hollandbloorview.ca/services/programs-services/youth-engagement/youth-advisory-council |
| Hôtel-Dieu Grace Healthcare Youth Advisory Council | https://www.hdgh.org/yac |
| Human Environments Analysis Laboratory (HEAL) Youth Advisory Council | http://theheal.ca/community/youth-advisory-council |
| IWK Youth Advisory Council | http://www.iwk.nshealth.ca/youth-advisory-council |
| Kids Help Phone National Youth Council | https://kidshelpphone.ca/get-involved/participate/national-youth-council |
| KidsAbility Youth Advisory Council | https://www.kidsability.ca/KAYAC |
| London Health Sciences Centre Children's Hospital Child and Youth Advisory Council | https://www.lhsc.on.ca/childrens-hospital/child-and-youth-advisory-council |
| McCreary Youth Advisory & Action Council | http://www.mcs.bc.ca/yac |
| Mental Health Commission of Canada (MHCC) Youth Council | https://www.mentalhealthcommission.ca/English/who-we-are/mhcc-networks/mhcc-youth-council |
| Métis Nation BC’s Métis Youth Mental Health and Wellness Initiative (formerly Métis Mental Health Youth Advisory Committee) | https://www.mnbc.ca/news-events/posts/mnbc-launches-m-tis-mental-health-youth-advisory-c |
| MICYRN KidsCan Young Persons’ Advisory Group | https://www.micyrn.ca/ypag |
| Mood Disorders Society of Canada National Youth Advisory Council | https://mdsc.ca/mood-disorders-society-of-canada-announces-new-national-youth-advisory-council |
| The New Mentality and Children’s Mental Health Ontario's Youth Action Committee | https://www.thenewmentality.ca/tnm-group/youth-action-committee/ |
| NorthBEAT Youth Advisory Group | http://northbeat.ca/northbeat-collab/northbeatyag |
| NorWest Co-op Community Health Youth Advisory Committee | https://norwestcoop.ca/get-involved/youth-advisory-committee |
| Ontario Centre of Excellence for Child & Youth Mental Health Youth Advisory Council | https://www.cymh.ca/en/about-us/youth-advisory-council.aspx |
| Pathstone Mental Health Youth Advisory Committee | https://pathstonementalhealth.ca/volunteer/advisory-committees |
| The Sandbox Project’s Young Canadians Roundtable on Health | http://sandboxproject.ca/about-the-ycrh |
| SickKids Children's Council | http://www.sickkids.ca/patient-family-resources/child-family-centred-care/Children-and-Families-as-Partners-in-Care/Childrens-Council/Childrens-Council.html |
| Stollery Youth Advisory Council | https://app.betterimpact.com/PublicOrganization/4730ee31-57b5-4d67-bdef-80184fbb2083/1 |
| YouthCan IMPACT Youth Advisory Group | http://youthcanimpact.com/engagement |
| Youth Mental Health Canada Youth Advisory Group | https://ymhc.ngo/about-us/yag-intro |
| Youth Wellness Hubs Ontario's Provincial Youth Advisory Council | https://frayme.ca/learning-institute-wrap/launching-youth-wellness-hubs-ontario%e2%80%99s-integrated-youth-service-provincial |
